# Conditioning adaptive combination of *P*-values method to analyze case-parent trios with or without population controls

**DOI:** 10.1038/srep28389

**Published:** 2016-06-24

**Authors:** Wan-Yu Lin, Yun-Chieh Liang

**Affiliations:** 1 Institute of Epidemiology and Preventive Medicine, College of Public Health, National Taiwan University, Taipei, Taiwan; 2 Department of Public Health, College of Public Health, National Taiwan University, Taipei, Taiwan

## Abstract

Detection of rare causal variants can help uncover the etiology of complex diseases. Recruiting case-parent trios is a popular study design in family-based studies. If researchers can obtain data from population controls, utilizing them in trio analyses can improve the power of methods. The transmission disequilibrium test (TDT) is a well-known method to analyze case-parent trio data. It has been extended to rare-variant association testing (abbreviated as “rvTDT”), with the flexibility to incorporate population controls. The rvTDT method is robust to population stratification. However, power loss may occur in the conditioning process. Here we propose a “conditioning adaptive combination of *P*-values method” (abbreviated as “conADA”), to analyze trios with/without unrelated controls. By first truncating the variants with larger *P*-values, we decrease the vulnerability of conADA to the inclusion of neutral variants. Moreover, because the test statistic is developed by conditioning on parental genotypes, conADA generates valid statistical inference in the presence of population stratification. With regard to statistical methods for next-generation sequencing data analyses, validity may be hampered by population stratification, whereas power may be affected by the inclusion of neutral variants. We recommend conADA for its robustness to these two factors (population stratification and the inclusion of neutral variants).

Investigating rare causal variants [minor allele frequency (MAF) < 1%] can help uncover the etiology of complex diseases^1^,^2^,^3^,^4^. With the development of next-generation sequencing technologies, some rare variants were found to be associated with complex human diseases^5^,^6^. Genetic epidemiology studies can be population-based, in which unrelated cases and controls are recruited, or family-based, in which genetically-related family members are enlisted. Recruiting case-parent trios is a simple and popular study design in family-based studies. If researchers can also obtain data from population controls, utilizing them in trio analyses can improve the power of statistical methods^7^.

The transmission disequilibrium test (TDT) is a well-known method used to analyze case-parent trios^8^,^9^. In TDT, only parents that are heterozygous at markers are informative. According to McNemar’s test^10^, alleles that are transmitted from parents to an affected child are compared to the alleles that are not transmitted. TDT has been extended to rare-variant association testing (referred to as “rvTDT” hereafter)^7^,^11^. Similar to the population data analysis methods^12^, rvTDT tests can be categorized as “burden-based tests” (or “linear combination tests”) or “kernel-based tests”. Jiang *et al*.^7^ further incorporated information from population controls to facilitate the discovery of rare causal variants. They proposed six tests: TLC(1), TLC(MAF), TLC(PC), TK(1), TK(MAF), and TK(PC). The former three are “burden-based tests” (or “linear combination tests”), and the latter three are “kernel-based tests”. The parenthetical expressions denote the weighting scheme on variants, where “1” represents unweighting, “MAF” represents inverse weighting by MAF according to parental genotypes, and “PC” represents weighting by population controls.

In addition to rvTDT, Schaid *et al*. proposed a burden test (referred to as “Burden” hereafter) and a kernel test (referred to as “Kernel” hereafter) as analysis methods for pedigrees, in which population controls can be included in analyses^13^. Their tests can analyze dichotomous or continuous traits by treating the traits as fixed and the genotypes as random. Because of this retrospective view, the tests are robust to the complicated ascertainment bias.

Depending on how information from multiple variants is aggregated, the above-mentioned rvTDT and the approaches proposed by Schaid *et al*. fall into two categories: burden-based tests and kernel-based tests. However, in next-generation sequencing data analyses, many neutral variants may be included in a functional region. The power of the above-mentioned tests may be diluted because of the inclusion of neutral variants. In contrast to burden-based methods that test the genetic score after summing variant counts within a region, the “adaptive combination of *P*-values method” (abbreviated as “ADA”) combines *P*-values of more significant variants^14^,^15^,^16^,^17^. ADA follows the *σ* − *MidP* method^18^ to assign MAF-related weights to rare variants when combining their *P*-values according to Fisher’s combination of *P*-values formula^19^. Moreover, ADA excludes larger *P*-values from the combination formula, based on the “truncated product method for combining *P*-values”^20^,^21^. However, because of the low frequencies and low statistical power of rare variants, determining a fixed *P*-value truncation threshold is not easy. ADA therefore considers variable *P*-value truncation thresholds during permutation, and adaptively finds the optimal threshold to truncate larger *P*-values that possibly arise from neutral variants. ADA has been extended to family data analysis^22^. However, that method^22^ is an unconditioning approach and thus is not robust to population stratification.

Depending on whether the test statistics are constructed by conditioning on parental genotypes, the above-mentioned methods can be categorized as conditioning approaches or unconditioning approaches. The rvTDT tests (including burden-based and kernel-based tests) and the family-based association test^23^ are conditioning approaches because they infer the distribution of offspring genotypes conditional on parental genotypes. Kernel and Burden tests^13^, and the previous ADA for family data analysis^22^, are unconditioning approaches. Conditioning approaches are robust to population stratification, but they usually lose some efficiency during the conditioning process. Unconditioning approaches are usually more powerful than conditioning approaches, but they may suffer from bias caused by population stratification.

To develop a powerful method that is also robust to population stratification, we propose a “conditioning adaptive combination of *P*-values method” (abbreviated as “conADA”). Because conADA adaptively finds the optimal threshold to truncate larger *P*-values that possibly arise from neutral variants, it is robust to the inclusion of neutral variants. This characteristic makes it more powerful than other conditioning approaches, such as the rvTDT tests^7^. Moreover, because its test statistic is developed by conditioning on parental genotypes, conADA generates valid statistical inference in the presence of population stratification. This property renders it more robust to population stratification than unconditioning approaches, such as the previous ADA for family data analysis^22^.

## Results

### Simulation study

#### Trios plus population controls

We used the Cosi program^24^, which is based on the coalescent process^25^, to simulate sequence data according to real human linkage disequilibrium (LD) patterns. To assess type-I error rates and the power of various statistical tests, a total of 10,000 data sets were generated. Each data set contained 20,000 haplotypes, and the length of each haplotype was 20 kb (kilo base pairs). Among the 20,000 haplotypes in each data set, 10,000 were simulated according to the LD patterns of Europeans, whereas the other 10,000 were simulated according to the LD patterns of Africans. Mimicking an exome-sequencing study, five subregions with a total length of ~5 kb were randomly selected from among the 20 kb haplotypes to represent the “captured coding sequences”. On average, the ~5 kb subregions contained ~87 variants with pooled MAF (in the pooled population of Europeans and Africans) ≤ 0.05. These ~87 variants formed the “analysis marker set” and were included in the analyses. When assessing type-I error rates, we assumed no causal loci existed in the region of interest.

When evaluating power, we considered two proportions of causal variants: “smaller” and “larger”. We specified 25% and 75% of rare variants (with pooled MAF ≤ 0.01) as causal variants, respectively.

We considered two situations for causal variants: (I) all were deleterious, (II) 50% were randomly chosen as deleterious, and the other 50% were protective. The per-locus population attributable fraction (PAF) was assumed to be 0.01 and 0.02 for situations (I) and (II), respectively. The statistical power is generally low for all methods under situation (II). For a meaningful comparison, we assigned a larger per-locus PAF to situation (II).

The genotypes of a subject were formed by randomly selecting two haplotypes from the haplotype pool. For a subject with a genotype-score vector ***G***, the probability of disease is 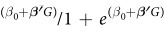. The possible elements in ***G*** were 0, 1, or 2, representing the numbers of minor alleles. Following the simulation setting in the rvTDT paper^7^, the intercept *β*_0_ was specified as 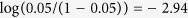 for Europeans and 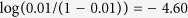 for Africans. This indicated that the disease prevalence in Europeans and Africans was assumed to be 0.05 and 0.01, respectively. The vector ***β*** incorporates the effects of variants on disease, and its *i*th element is 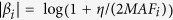, where *η* and *MAF*_*i*_ are the PAF and frequency of the *i*th variant, respectively. The setting of *β*_*i*_ s was derived from the relationship between the relative risk of exposure and PAF (see Appendix B of^7^). Supplemental Fig. S1 presented the distributions of *β*_*i*_ s for Europeans and Africans, when all causal variants were deleterious. In situation (II), 50% of causal variants were protective, and their *β*_*i*_ s were <0. A total of 500 case-parent trios and 500 (or 1000, 1500, 2000) unrelated controls were generated according to the above-mentioned model. The source populations for both trios and unrelated controls comprised 0:100 (all were Africans), 20:80, 50:50, 80:20, or 100:0 (all were Europeans) ratios of Europeans to Africans, respectively.

Furthermore, we evaluated the performance of all methods given a substantial difference in the source populations of trios and controls. Following Jiang *et al*.^7^, we assumed the trio-parent population comprised 80:20, 60:40, and 80:20 ratios of Europeans to Africans, and the control source population comprised 20:80, 40:60, and 60:40 ratios of Europeans to Africans.

Considering the smaller proportion of causal variants, 25% of rare variants (with pooled MAF ≤ 0.01) were randomly specified as causal. Although 25% was not low, most of the causal variants were not observed in the sample of 500 trios and 500 (or 1000, 1500, 2000) unrelated controls. In the African samples, ~4 causal loci (with pooled MAF ≤ 0.01) were observed in the analysis marker set that contained ~87 loci (with pooled MAF ≤ 0.05), and the proportion of observed causal variants in the analysis marker set was approximately 4.6% 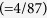. Because the European population showed less genetic diversity than the African population^26^, only ~2 causal loci were observed in the European samples.

Considering the larger proportion of causal variants, 75% of rare variants (with pooled MAF ≤ 0.01) were randomly specified as causal. Although 75% was a large percentage, most of the causal variants were not observed in the sample of 500 trios and 500 (or 1000, 1500, 2000) unrelated controls. In the African samples, ~12 causal loci (with pooled MAF ≤ 0.01) were observed in the analysis marker set that contained ~87 loci (with pooled MAF ≤ 0.05), and the proportion of causal variants in the analysis marker set was approximately 13.8% 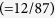. In the European samples, only ~6 causal loci were observed. We summarized the setting of the “smaller” and “larger” proportions of causal variants in Supplemental Table S1.

#### Trios only

In the second part of our simulation study, we only analyzed the 500 trios, mimicking situations in which population controls cannot be obtained. The trio-parent population comprised 0:100 (all were Africans), 20:80, 50:50, 80:20, or 100:0 (all were Europeans) ratios of Europeans to Africans, respectively. Similarly, we considered the two situations for causal variants: (I) all were deleterious, (II) 50% were specified as deleterious, and the other 50% were protective. The per-locus PAF was assumed to be 0.01 and 0.02 for situations (I) and (II), respectively. The statistical power is generally low for all methods under situation (II). For a meaningful comparison, we assigned a larger per-locus PAF to situation (II).

### Tests under comparison

#### Trios plus population controls

We let conADA(PC) be the conADA method for analyzing trios with population controls. In addition to conADA(PC), the tests under comparison included TLC(1), TLC(MAF), TLC(PC), TK(1), TK(MAF), TK(PC)^7^, Burden, Kernel^13^, SKAT^27^, SKAT-O^28^, and ADA^14^. The last three methods were originally proposed to analyze unrelated cases and controls; therefore, only the affected offspring in trios (each trio contributed one case) and the unrelated controls were used in analyses. The R packages rvTDT-1.0^7^, pedgene-2.1^13^, and SKAT-1.0.1^28^, and the R code of ADA ( http://homepage.ntu.edu.tw/~linwy/ADA.r) were used to implement these statistical tests. The *P*-value of ADA was assessed with 1,000 permutations. The *P*-value of conADA was obtained with the above-mentioned sequential Monte Carlo permutation^29^ (the minimum and maximum numbers of permutations were set as 100 and 10000, respectively). Following the default settings in the R packages SKAT^27^,^28^, pedgene^13^, and rvTDT [MAF weighting: TLC(MAF) and TK(MAF)]^7^, the weight given to the *j*th genetic variant was *Beta*(*MAF*_*j*_;1,25), where *MAF*_*j*_ was the frequency of the *j*th variant. The *P*-values of SKAT and SKAT-O were obtained by the Davies method^30^.

#### Trios only

We let conADA(MAF) be the conADA method for analyzing trios without population controls. When only trios were analyzed, TLC(PC) and TK(PC)^7^, SKAT^27^, SKAT-O^28^, and ADA^14^ could not be performed. Therefore, we compared conADA(MAF) with TLC(1), TLC(MAF), TK(1), TK(MAF)^7^, Burden, and Kernel^13^. The *P*-value of conADA(MAF) was assessed with the above-mentioned sequential Monte Carlo permutation method^29^ (the minimum and maximum numbers of permutations were set as 100 and 10000, respectively). For all methods except TLC(1) and TK(1), the weight given to the *j*th genetic variant was *Beta*(*MAF*_*j*_;1,25), where *MAF*_*j*_ was the frequency of the *j*th variant.

### Type-I error rates

By setting the PAF at exactly 0% for all variants, we assessed type-I error rates with 10,000 simulated data sets.

#### Trios plus population controls

When the ethnicity compositions in trios and in population controls were the same: When the ethnicity compositions in trios and in unrelated controls were the same, all the 12 tests were valid; their type-I error rates matched the nominal significance levels (Supplemental Table S2). In that table, we also listed the type-I error rates of conADA with 21 *P*-value truncation thresholds, i.e., *θ*_1_ = 0.05, *θ*_2_ = 0.06, 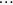, *θ*_21_ = 0.25. The results for 21 thresholds were similar to those for 11 thresholds (*θ*_1_ = 0.10, *θ*_2_ = 0.11, 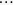, *θ*_11_ = 0.20).

When the ethnicity compositions in trios and in population controls were NOT similar: Given a substantial difference in the source populations of trios and controls, only seven conditioning approaches were valid and their type-I error rates were listed in Supplemental Table S3. The other five tests including Kernel, Burden^13^, SKAT^27^, SKAT-O^28^, and ADA^14^ had very large type-I error rates (~70–100%). Because these five tests were invalid in this scenario, we did not add their results to Supplemental Table S3.

#### Trios only

When unrelated controls could not be obtained, Supplemental Table S4 showed that all the seven tests for trios {TLC(1), TLC(MAF), TK(1), TK(MAF)^7^, Burden, Kernel^13^, conADA(MAF)} were valid.

### Power comparisons

#### Trios plus population controls

**Figure 1:**
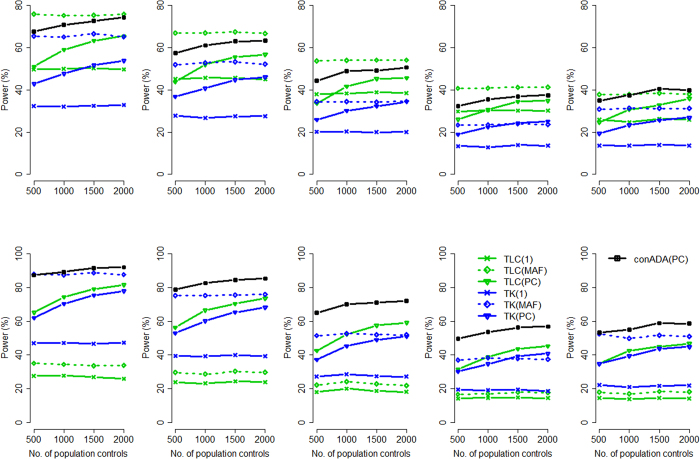
Power comparison given the same source population for trios and controls (larger proportion of causal variants). The figure shows the empirical power given the nominal significance level of 0.05. Top row: all causal variants were deleterious; bottom row: 50% of causal variants were deleterious and 50% were protective. The *x*-axis is the number of population controls, whereas the *y*-axis is the power. The study subjects (including trio members and population controls) comprised 0:100 (the left column, all were Africans), 20:80 (the second column), 50:50 (the middle column), 80:20 (the fourth column), and 100:0 (the right column, all were Europeans) ratios of Europeans to Africans, respectively.

When the ethnicity compositions in trios and in population controls were the same: Given a larger proportion of causal variants: When the ethnicity compositions in trios and in unrelated controls were the same, Fig. 1 presents the power of the seven conditioning approaches, considering a larger proportion of causal variants. Supplemental Tables S5 and S6 show the power of all the 12 tests in this situation, when all causal variants were deleterious and when 50% of causal variants were deleterious and 50% were protective, respectively. In Supplemental Table S5, we also list the power of conADA with 21 *P*-value truncation thresholds, i.e., *θ*_1_ = 0.05, *θ*_2_ = 0.06, 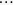, *θ*_21_ = 0.25. Its performance was very similar to conADA with 11 thresholds. Therefore, we only used 11 *P*-value truncation thresholds (*θ*_1_ = 0.10, *θ*_2_ = 0.11, 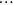, *θ*_11_ = 0.20) for conADA hereafter.

With an increasing number of population controls, all tests had increasing power, except TLC(1), TLC(MAF), TK(1), and TK(MAF). These four tests did not utilize the information from population controls; therefore, their performance did not vary with the number of controls. Comparing the burden-based (or linear combination) tests [TLC(1), TLC(MAF), TLC(PC)] and the kernel-based tests [TK(1), TK(MAF), TK(PC)] from the rvTDT package^7^, the burden-based tests outperformed the kernel-based tests given the same weighting scheme, when all causal variants were deleterious (top row of Fig. 1). When 50% of causal variants were deleterious and 50% were protective (bottom row of Fig. 1), the kernel-based tests were more powerful than the burden-based tests, except the PC weighting scheme.

Among the six rvTDT tests, in general, TLC(MAF) had the highest power when all causal variants were deleterious (top row of Fig. 1). This was because we here specified 75% of rare variants (with pooled MAF ≤ 0.01) as causal variants, and MAF therefore became a good index for causal variants. When 50% of causal variants were deleterious and 50% were protective, TK(MAF) was the most powerful test among the six rvTDT tests. However, TLC(PC) outperformed TK(MAF) given a larger number of population controls (bottom row of Fig. 1).

Considering different weighting schemes in rvTDT, MAF weighting [TLC(MAF) and TK(MAF)] was more powerful than unweighting [TLC(1) and TK(1)]. In reality, scientists have found that the majority of functional variants are very rare (MAF < 0.5%) and that rare variants are more likely to have larger predicted functional impacts than common variants^31^. Therefore, assigning weights according to MAFs is justifiable and powerful. PC weighting [TLC(PC) and TK(PC)] was even more powerful than MAF weighting, when the number of controls was sufficiently large.

Considering the seven conditioning tests, conADA(PC) given a larger number of PC had comparable power with TLC(MAF) when all causal variants were deleterious (top row of Fig. 1), and it outperformed all the rvTDT tests when 50% of causal variants were deleterious and 50% were protective (bottom row of Fig. 1).

Considering all the 12 tests (Supplemental Tables S5 and S6), in general, ADA^14^ was the most powerful test when all the causal variants were deleterious (Supplemental Table S5), whereas Kernel^13^ performed best when 50% of causal variants were deleterious and 50% were protective (Supplemental Table S6). Note that SKAT-O^28^ is a linear combination of SKAT statistic^27^ and the burden-based test statistic. When 50% causal variants were deleterious and 50% were protective, the burden-based test suffered from a power loss^12^; therefore, it is not surprising that SKAT-O was inferior to SKAT in this situation (Supplemental Table S6).

It is worth mentioning that tests analyzing Africans presented higher power than those analyzing Europeans (in Fig. 1, generally, power was decreasing from the left column to the right column). This was because the African population showed greater genetic diversity than the European population^26^, and ~12 causal loci were observed in African samples, whereas only ~6 causal loci were observed in European samples (see Supplemental Table S1).

**Figure 2:**
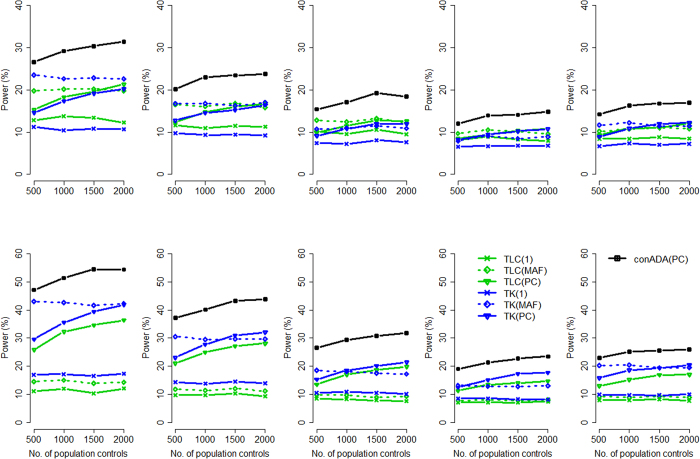
Power comparison given the same source population for trios and controls (smaller proportion of causal variants). The figure shows the empirical power given the nominal significance level of 0.05. Top row: all causal variants were deleterious; bottom row: 50% of causal variants were deleterious and 50% were protective. The *x*-axis is the number of population controls, whereas the *y*-axis is the power. The study subjects (including trio members and population controls) comprised 0:100 (the left column, all were Africans), 20:80 (the second column), 50:50 (the middle column), 80:20 (the fourth column), and 100:0 (the right column, all were Europeans) ratios of Europeans to Africans, respectively.

Given a smaller proportion of causal variants: In this scenario, we specified 25% of rare variants (with pooled MAF ≤ 0.01) as causal variants. Figure 2 presents the power of the seven conditioning approaches, and Supplemental Tables S7 (when all causal variants were deleterious) and S8 (when 50% of causal variants were deleterious and 50% were protective) show the power of all the 12 tests.

Given the smaller proportion of causal variants, conADA(PC) became the most powerful test among the seven conditioning tests (Fig. 2) because more neutral variants had to be truncated in this situation. Considering all the 12 tests (Supplemental Tables S7 and S8), in general, ADA^14^ was the most powerful test when all the causal variants were deleterious (Supplemental Table S7), and Kernel^13^ performed best when 50% of causal variants were deleterious and 50% were protective (Supplemental Table S8).

Among the six rvTDT tests^7^, the kernel-based tests [TK(1), TK(MAF), TK(PC)] performed better than the burden-based tests [TLC(1), TLC(MAF), TLC(PC)] given the same weighting scheme, when 50% of causal variants were deleterious and 50% were protective (bottom row of Fig. 2). Among the six rvTDT tests, TK(MAF) and TK(PC) had higher power. TK(PC) outperformed TK(MAF) given a larger number of PC.

Table 1 lists the average computation time for 500 trios and 500 (or 1000, 1500, 2000) population controls. Because the *P*-values of conADA(PC) were obtained from 100–10,000 permutations, it was the most computationally intensive method. We also listed the average computation time for conADA(PC) based on 10–1000 permutations. When the nominal significance level was specified as 0.01 or 0.05, conADA(PC) with 10–1000 permutations was valid in the sense that its type-I error rates matched the nominal significance levels (results not shown). In this situation, using 10–1000 permutations for conADA(PC) would save a significant amount of time.

**Figure 3:**
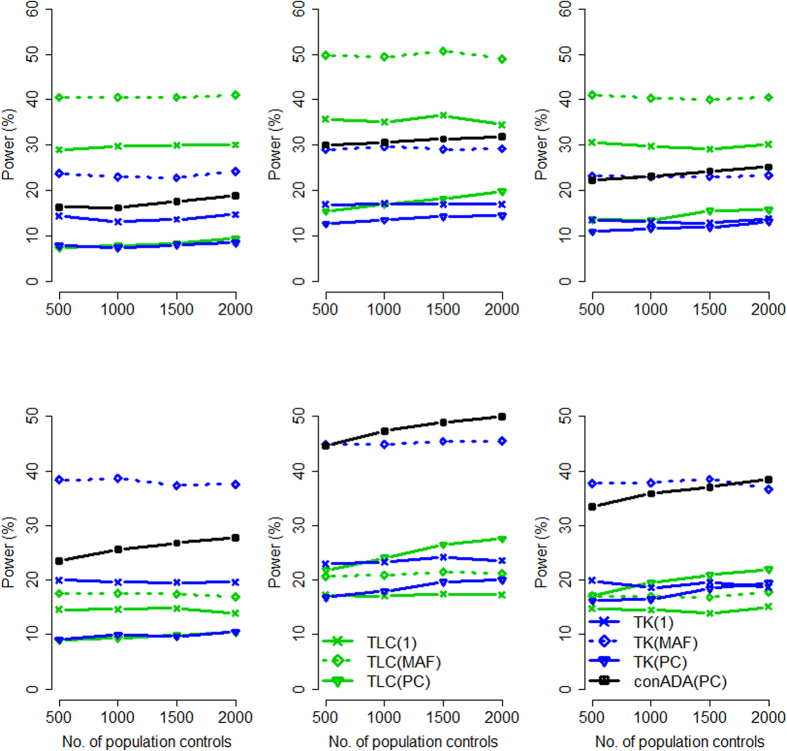
Power comparison given a substantial difference in the source populations of trios and controls (larger proportion of causal variants). The figure shows the empirical power given the nominal significance level of 0.05. Top row: all causal variants were deleterious; bottom row: 50% of causal variants were deleterious and 50% were protective. The *x*-axis is the number of population controls, whereas the *y*-axis is the power. The trio-parent population comprised 80:20 (left column), 60:40 (middle column), and 80:20 (right column) ratios of Europeans to Africans, and the control source population comprised 20:80 (left column), 40:60 (middle column), and 60:40 (right column) ratios of Europeans to Africans.

When the ethnicity compositions in trios and in population controls were NOT similar: Given a larger proportion of causal variants: When the source populations of trios and controls were not identical, only the seven conditioning tests were valid; thus, only they were considered in power comparisons. Figure 3 presents their power given a larger proportion of causal variants. TLC(MAF) had the highest power when all causal variants were deleterious (top row of Fig. 3). Because the ethnicity compositions in trios and in population controls were not similar, tests weighted by PC all suffered from a power loss, especially for the left column of Fig. 3 (where the ethnicity compositions in trios and in population controls were very different). TLC(PC) and TK(PC) presented low power because of this inappropriate weighting scheme. Although conADA(PC) was also weighted by population controls, it outperformed TLC(PC) and TK(PC) because of its truncation of neutral variants.

**Figure 4:**
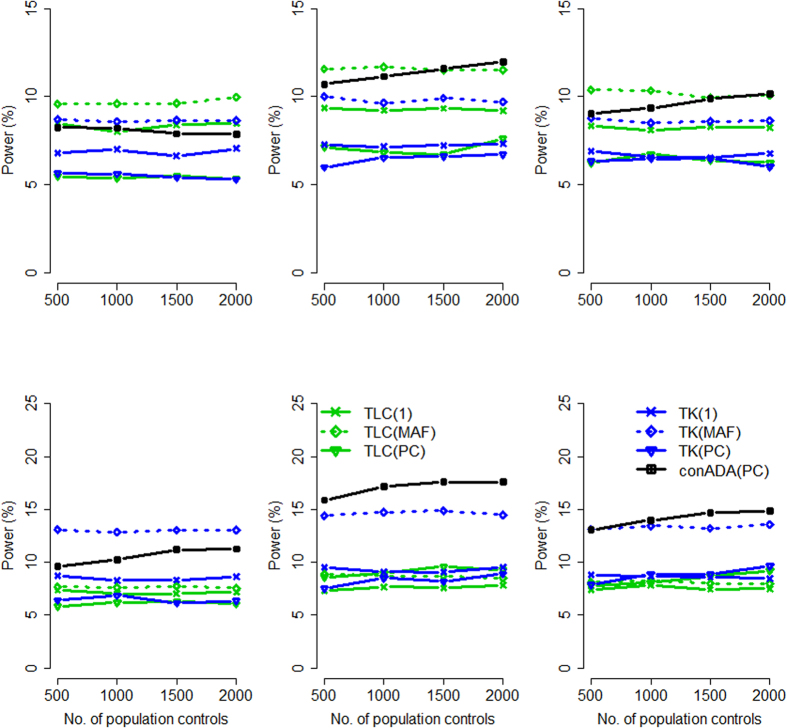
Power comparison given a substantial difference in the source populations of trios and controls (smaller proportion of causal variants). The figure shows the empirical power given the nominal significance level of 0.05. Top row: all causal variants were deleterious; bottom row: 50% of causal variants were deleterious and 50% were protective. The *x*-axis is the number of population controls, whereas the *y*-axis is the power. The trio-parent population comprised 80:20 (left column), 60:40 (middle column), and 80:20 (right column) ratios of Europeans to Africans, and the control source population comprised 20:80 (left column), 40:60 (middle column), and 60:40 (right column) ratios of Europeans to Africans.

Given a smaller proportion of causal variants: Figure 4 presents the power of the seven conditioning tests given a smaller proportion of causal variants. When the ethnicity compositions in trios and in population controls were moderately different, conADA(PC) could still be a relatively more powerful test among the seven conditioning tests (see the middle and right columns of Fig. 4). However, when the ethnicity compositions in trios and in population controls were substantially different (see the left column of Fig. 4), the advantage of conADA(PC) from truncating neutral variants could not overcome the inappropriate PC weighting.

#### Trios only

**Figure 5:**
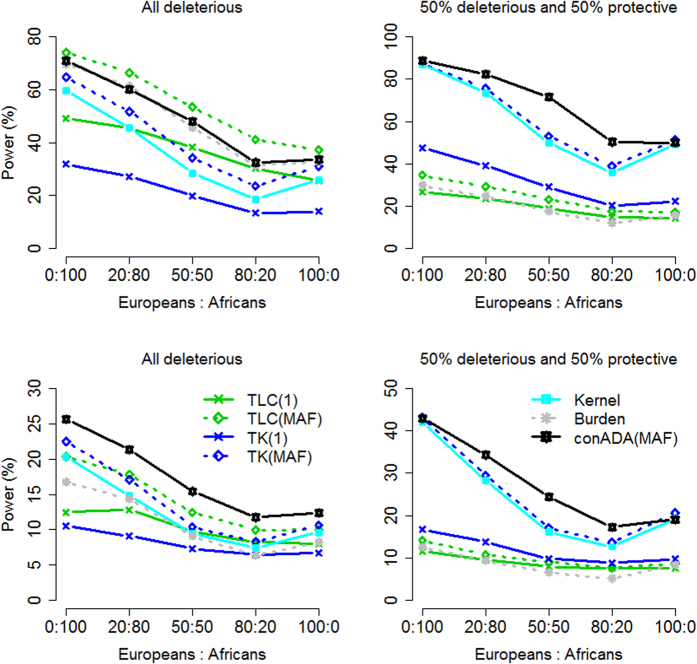
Power comparison when only trios could be obtained (without population controls). The figure shows the empirical power given the nominal significance level of 0.05. Left column: all causal variants were deleterious; right column: 50% of causal variants were deleterious and 50% were protective. Top row: a larger proportion of causal variants; bottom row: a smaller proportion of causal variants. The *x*-axis represents the ethnicity composition in trios, and the *y*-axis shows the power.

Figure 5 presents the power of the seven tests that can analyze trios without PC. TLC(MAF) had the highest power when all causal variants were deleterious and when the proportion of causal variants was larger (upper left of Fig. 5). In other situations, our conADA(MAF) was generally the most powerful method.

Comparing the burden-based tests {or “linear combination tests”, such as TLC(1), TLC(MAF)^7^, and Burden^13^} with the kernel-based tests {such as TK(1), TK(MAF)^7^, and Kernel^13^}, burden-based tests outperformed the corresponding kernel-based tests when all causal variants were deleterious and when the proportion of causal variants was larger (upper left of Fig. 5). When 50% of causal variants were deleterious and 50% were protective, the kernel-based tests were consistently more powerful than the burden-based tests (see the right column of Fig. 5).

### Application to Genetic Analysis Workshop (GAW) 18 Data

GAW 18 data sets contained 20 Mexican-American pedigrees that were selected from two San Antonio-based family studies: SAFHS^32^ and SAFDGS^33^,^34^. Here we focused on the dichotomous hypertension status at baseline. Hypertension was defined as systolic blood pressure (SBP) > 140, diastolic blood pressure (DBP) > 90, or on antihypertensive medications at the examination^35^. Hypertension affects up to 30% of adults in Western countries, and it is a major risk factor for kidney disease, stroke, and coronary heart disease^36^.

There were 21 case-parent trios and 90 unrelated controls extracted from the 20 pedigrees. Although these 90 unrelated controls were also drawn from the 20 Mexican-American pedigrees, they were genetically unrelated to each other or to the members of the 21 trios. Formal assessment of the relatedness was done with the function “pedigree.unrelated” in the R package “kinship2” (version 1.6.4)^37^.

In the GAW18 data sets, only genotypes for odd numbered chromosomes were distributed. Following Feng and Zhu^38^, we grouped variants into genes or regions according to the Ensembl software ( http://www.ensembl.org). Totally, there were 38,091 genes or regions. Similar to our simulation study, the analysis marker set for each gene/region was formed by including all the variants with MAF ≤ 0.05.

From our simulation study, we found that the seven conditioning tests [including the six rvTDT tests and conADA(PC)] were valid even when the ethnicity compositions in trios and in population controls were not similar. Therefore, we analyzed the GAW 18 data with these seven robust tests. The R package rvTDT-1.0^7^ was used to perform the six rvTDT tests. The *P*-value of conADA(PC) was obtained with the sequential Monte Carlo permutation^29^, in which the minimum and maximum numbers of permutations were set as 10^2^ and 10^6^, respectively. The 11 candidate *P*-value truncation thresholds were *θ*_1_ = 0.10, *θ*_2_ = 0.11, 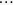, and *θ*_11_ = 0.20. The R code to analyze the GAW 18 data set can be downloaded from http://homepage.ntu.edu.tw/~linwy/conADA.r.

**Figure 6:**
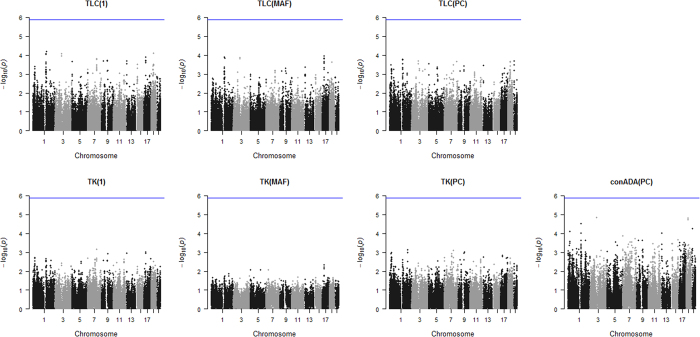
Results for Genetic Analysis Workshop 18 (GAW18) data. The figure shows the results of the seven tests when analyzing the variants with MAF ≤ 0.05 in the GAW18 data. The *x*-axis is the chromosome number, and the *y*-axis is –log_10_(*P*-value). The *P*-value of conADA(PC) was obtained with the sequential Monte Carlo permutation, and the minimum and maximum numbers of permutations were set as 10^2^ and 10^6^, respectively. Because there were totally 38,091 genes/regions, the significance level was set at 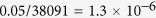, marked at 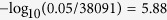 with blue lines.

Figure 6 shows the Manhattan plots based on the seven tests, respectively. Because there were totally 38,091 genes/regions, the significance level was set at 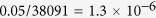, marked at 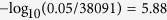 with blue lines. For each of the seven tests, no genes/regions achieved significance after correcting for multiple comparisons.

Supplemental Fig. S2 presents the Manhattan plots when only variants with MAF ≤ 0.01 were analyzed. Compared with the results for variants with MAF ≤ 0.05 (Fig. 6), the significance was generally weakened when only variants with MAF ≤ 0.01 were considered.

## Discussion

The conADA method truncates variants with larger *P*-values. We define singletons (doubletons) as alleles present only once (twice) in a data set. In conADA, after truncating variants with larger *P*-values, no singletons were left in any simulation data set, whereas ~6.2% of the remaining variants were doubletons. The conADA method removed all singletons from the analysis because of their larger *P*-values. Therefore, this method cannot detect causal variants that appear as singletons in a data set. In reality, it is difficult to distinguish these causal variants from random genetic variation, and accurate functional predictions of such variants are important to prioritize them as likely to be causal^39^.

Case-parent association tests are robust to the bias introduced by population substructure. If unrelated controls (from the same source population of trios) can be obtained, utilizing them in rare-variant case-parent association studies can enhance the power of statistical methods, such as TK(PC) and TLC(PC) in the rvTDT package^7^, and Kernel and Burden in the pedgene package^13^. However, when the controls are not from the same source population of trios, TK(PC) and TLC(PC) suffer from a power loss, whereas Kernel and Burden are even invalid. Our conADA(PC) is less vulnerable in this situation, both in terms of validity (compared with Kernel and Burden in the pedgene package^13^) and power {compared with TK(PC) and TLC(PC) in the rvTDT package^7^}.

The conADA(PC) method adaptively combines association signals of variants with smaller *P*-values, which are more likely to be causal. The optimal *P*-value truncation threshold is searched through permutations, and therefore requires more computation time. The strength of conADA(PC) is its robustness to population stratification and the inclusion of neutral variants. However, the weakness is its longer computation time compared with other methods. Because analytical *P*-values cannot be obtained, it is time-consuming to perform conADA given a genome-wide significance level (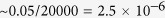). A reasonable two-stage strategy is to first scan the whole genome with 10–1000 permutations, and then increase the number of permutations to meet the genome-wide significance level for the more significant genes.

As mentioned by Feng and Zhu^38^, the presence of admixture and LD among rare variants imposes challenges on family-based analyses. Although rare variants are likely independent in general^40^, Feng and Zhu^38^ have found that substantial LD among rare variants can be introduced by population admixture. To study the effect of LD on the performance of association tests, we also followed Jiang *et al*.^7^ to simulate admixture. We sampled 2,000 controls from a source population in which individuals have an average admixture proportion of 80% African and 20% European, and 500 trios were sampled from a parent population in which individuals have an average admixture proportion of 20% African and 80% European. Prevalence was set at 1% and 5% for the control source population and the trio source population, respectively. Similar to the previous simulation section, variants with MAF ≤ 0.05 were used for analyses. Supplemental Table S9 lists the Type I error rates and statistical power for the lower LD and higher LD scenarios, respectively.

Although TLC(PC), TK(PC), and conADA(PC) used the population controls with a very different admixture proportion from that in trios, they maintained test sizes even in such an extreme admixture scenario (Jiang *et al*.^7^ have presented the validity of TLC(PC) and TK(PC)). From the power results in Supplemental Table S9, we find that conADA(PC) has a larger power in lower LD scenarios than in higher LD scenarios. This is because truncation of neutral variants is more advantageous when these variants have little correlations with causal variants.

## Methods

Let 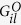, 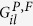, and 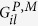 be the offspring, paternal, and maternal genotype scores (0, 1, or 2, representing the number of minor alleles), respectively, at the *l*th variant in the *i*th trio (*i* = 1, 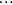, *n*, *l* = 1, 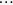, *L*). Let 
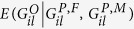
 represent the expected offspring genotype score conditional on parental genotypes at the *l*th variant of the *i*th trio. Under the null hypothesis of no association between the *l*th variant and disease, the test statistic


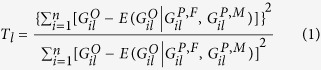


asymptotically follows *χ*^2^ distribution with 1 degree of freedom.

Consider there are *L* loci in the chromosomal region of interest. We calculate the statistic in equation (1) and obtain the *P*-values *p*_1_, *p*_2_, 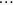, *p*_*L*_ for the *L* loci, respectively. In conADA, we exclude markers with larger *P*-values, which are more likely to be neutral. The optimal *P*-value truncation threshold is determined by permutation. We consider 11 candidate *P*-value truncation thresholds, *θ*_1_ = 0.10, *θ*_2_ = 0.11, 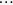, *θ*_11_ = 0.20. Using a wider range of *P*-value truncation thresholds, for example, *θ*_1_ = 0.05, *θ*_2_ = 0.06, 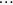, *θ*_21_ = 0.25, will not contribute a noticeable power gain to conADA (shown in simulation results). Summarizing the markers with *P*-values smaller than *θ*_*j*_ (the *j*th truncation threshold), the significance score is


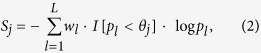


where *I* [·] is an indicator variable coded as 1 or 0, and *w*_*l*_ is the weight given to the *l*th marker.

When population controls cannot be obtained, the weight given to the *l*th marker is set at *Beta* (*MAF*_*l*_; 1, 25), where *MAF*_*l*_ is the variant frequency for the *l*th marker calculated from parental genotypes. The Beta density function with the two parameters 1 and 25 is the default weighting scheme of SKAT and SKAT-O^27^,^28^, pedgene (the R package to implement Kernel and Burden^13^), and rvTDT using the MAF weighting scheme [i.e., TLC(MAF) and TK(MAF)]^7^.

When population controls can be obtained, and the genotypes of the controls and the parents of trios are as summarized in Table 2, the weights *w*_*l*_s (*l* = 1, 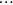, *L*) in Eq. (2) are specified as:


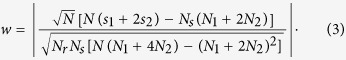


This weighting scheme is based on the Armitage trend test^41^. If the genotype distributions of parents in trios and population controls are very different, this weight will be large. We let conADA(PC)/conADA(MAF) be the conADA method for analyzing trios with/without population controls, respectively.

Then we use the sequential Monte Carlo permutation^29^ to quantify *P*-values. Like most rare variant association tests, conADA is used to analyze a gene or a small chromosomal region. We therefore assume no recombination occurs within the region of interest. As mentioned by Zhang *et al*.^42^, under the assumption of no recombination, each haplotype can be regarded as an allele at a single multiallelic locus. We then follow the permutation test proposed by Fan *et al*.^43^. For each trio in a permutation, we only consider two events regarding “haplotype transmission” (or “allelic transmission”, because a haplotype is regarded as an allele under the assumption of no recombination): the offspring has *observed diplotype* vs. the offspring has *unobserved diplotype*. The occurrence of *observed diplotype* means the offspring has the two haplotypes originally transmitted from the parents, one from the father and the other from the mother. The occurrence of *unobserved diplotype* means the offspring has the two originally non-transmitted haplotypes, one belonging to the father and the other belonging to the mother. Under the null hypothesis, the offspring has a probability of 1/2 to exhibit the *observed diplotype* or *unobserved diplotype*. For a set of *n* trios, there are 2^*n*^ enumerations in total^43^. When performing permutations, we randomly toss a fair coin to assign *observed diplotype* or *unobserved diplotype* to each child. If a child is selected to have the *observed diplotype*, his/her permuted genotype scores will remain the same as his/her unpermuted genotype scores. If he/she is selected to have the *unobserved diplotype*, his/her permuted genotype score at the *l*th variant will be


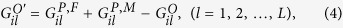


where 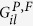 and 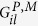 are the genotype scores of the father and the mother, respectively, and 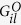 is the unpermuted offspring genotype score.

If we perform *B* permutations, we compare *S*_*j*_ (the significance score under the *j*th *P*-value truncation threshold) with 
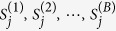
 (the significance scores under the same *P*-value truncation threshold for the *B* permuted samples), and the *P*-value corresponding to *S*_*j*_ is 
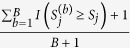
. Across the 11 thresholds, the minimum *P*-value for the observed sample (*MinP*) is compared with the minimum *P*-values for the *B* permuted samples (*MinP*^(1)^, *MinP*^(2)^, 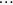, *MinP*^(*B*)^), and the “adjusted *P*-value” is calculated as 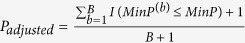. The sequential Monte Carlo permutation^29^ is used to decrease the computation time. We let the minimum and maximum numbers of permutations be *B*_min_ = 100 and *B*_max_ = 10000, respectively. The permutation process will be terminated if 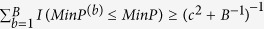 or if *B* = *B*_max_, and it will not be terminated if *B* < *B*_min_. Following Besag and Clifford^29^, we specified *c* = 0.25, meaning that the standard error of *P*_*adjusted*_ is approximately 25% of the *P*-value.

## Additional Information

**How to cite this article**: Lin, W.-Y. and Liang, Y.-C. Conditioning adaptive combination of *P*-values method to analyze case-parent trios with or without population controls. *Sci. Rep.* **6**, 28389; doi: 10.1038/srep28389 (2016).

## Supplementary Material

Supplementary Information

## Figures and Tables

**Table 1:** Average computation time (in seconds) for 500 trios and 500, 1000, 1500, or 2000 population controls.

No. of population controls	rvTDT	pedgene	SKAT^a^	ADA^b^	conADA(PC)^c^	conADA(PC)^d^
**500**	15.68	4.15	27.60	62.52	76.50	486.64
**1000**	15.36	6.04	39.61	81.27	78.58	490.72
**1500**	15.31	8.53	2.51	99.48	73.43	487.79
**2000**	15.21	11.82	3.25	116.09	77.12	492.15

^a^According to the default of the SKAT package, a small sample adjustment is applied when the total sample size is <2000. Therefore, in addition to 500 cases contributed by 500 trios, when the number of population controls is <1500, the total sample size is <2000 and SKAT required more time.

^b^The *P*-values of ADA were obtained with 1,000 permutations.

^c^The *P*-values of conADA were obtained with the sequential Monte Carlo permutation^29^, and the minimum and maximum numbers of permutations were set as 10 and 1000, respectively.

^d^The *P*-values of conADA were obtained with the sequential Monte Carlo permutation^29^, and the minimum and maximum numbers of permutations were set as 100 and 10,000, respectively.

**Table 2:** The 2 × 3 contingency table summarizing trio parents’ and controls’ genotypes.

Number of minor alleles	0	1	2	Total
Population controls	*r*_0_	*r*_1_	*r*_2_	*N*_*r*_
Trio parents	*s*_0_	*s*_1_	*s*_2_	*N*_*s*_
Total	*N*_*0*_	*N*_*1*_	*N*_*2*_	*N*
