# Supplementary material for: Conditioning adaptive combination of P-values method to analyze case-parent trios with or without population controls
Source: Sci Rep. 2016 Jun 24;6:28389. doi: 10.1038/srep28389 (PMC4920030; doi:10.1038/srep28389)
Supplement: Supplementary Information [file srep28389-s1.pdf]

Conditioning adaptive combination of *P*-values method to analyze case-parent trios with or without population controls

Wan-Yu Lin [1,2]*, Yun-Chieh Liang [1]

[1] Institute of Epidemiology and Preventive Medicine, College of Public Health, National Taiwan University, Taipei, Taiwan

[2] Department of Public Health, College of Public Health, National Taiwan University, Taipei, Taiwan

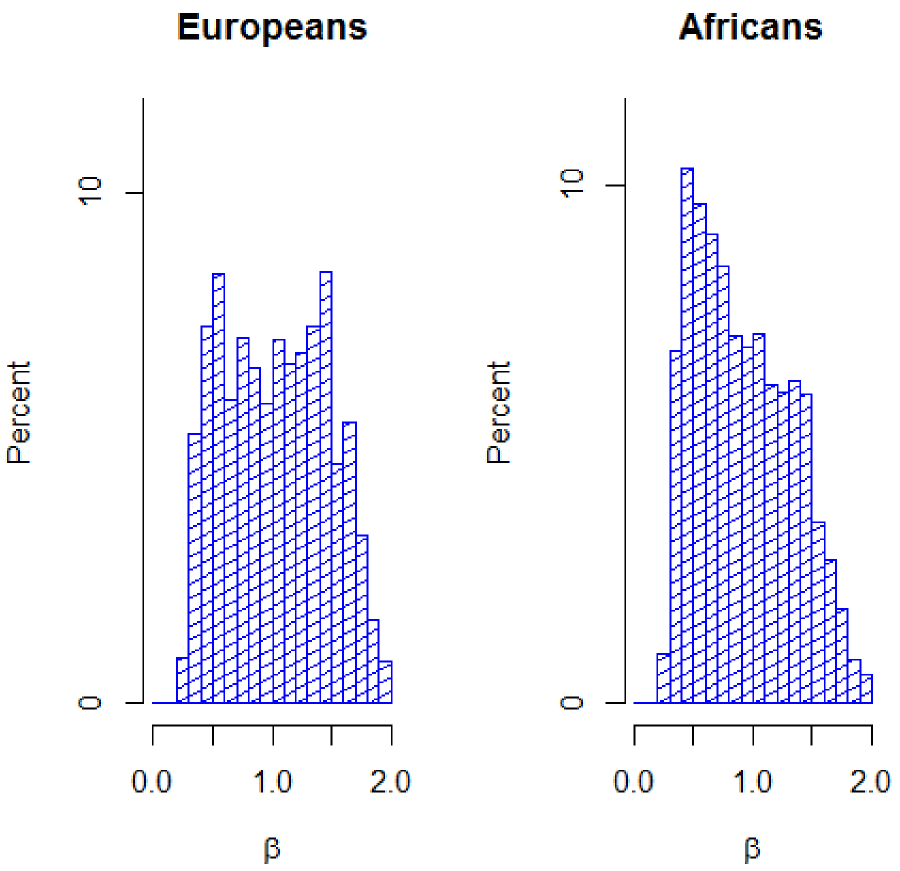

Supplemental Figure S1: Distributions of β_is for Europeans and Africans when all causal variants were deleterious

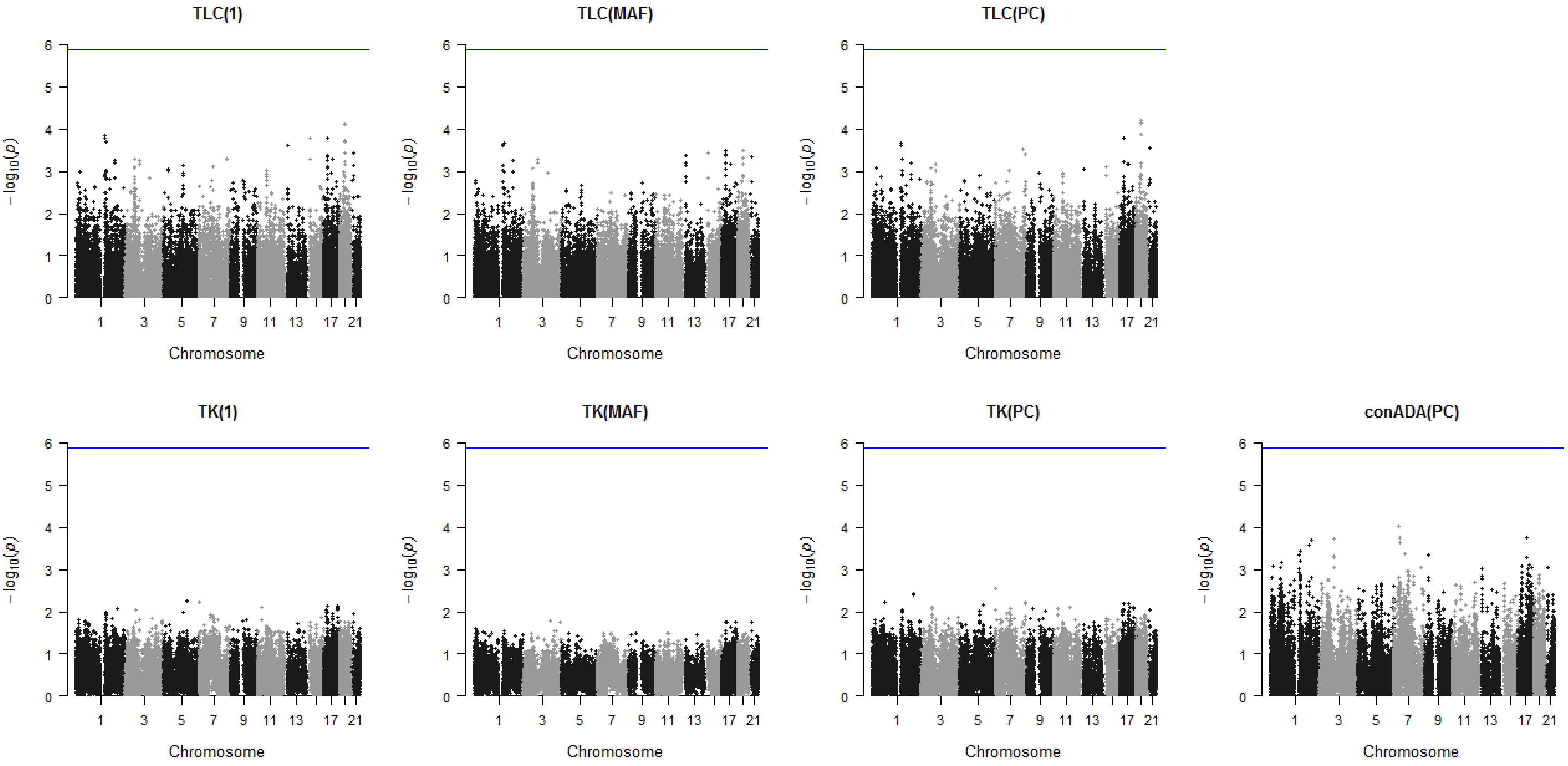

Supplemental Figure S2: Results for Genetic Analysis Workshop 18 (GAW18) data. The figure shows the results of the seven tests when analyzing the variants with MAF$\leq$0.01 in the GAW18 data. The *x*-axis is the chromosome number, and the *y*-axis is $-\log_{10}$(*P*-value). The *P*-value of conADA(PC) was obtained with the sequential Monte Carlo permutation, and the minimum and maximum numbers of permutations were set as 10^2 and 10^6, respectively. Because there were totally 38,091 genes/regions, the significance level was set at $0.05/38091 = 1.3\times10^{-6}$, marked at $-\log_{10}\left(0.05/38091\right) = 5.88$ with blue lines.

**Proportion of causal variants**		**“smaller”**	**“larger”**
Percentage in rare variants (pooled MAF $\leq$ 0.01)		25%	75%
The number of causal loci observed in a simulation sample	**Africans**	~4	~12
	**Europeans**	~2 [a]	~6
The proportion of observed causal variants in the analysis marker set (the analysis marker set contained ~87 variants with pooled MAF $\leq$ 0.05)	**Africans**	$\sim 4/87 = 4.6\%$	$\sim 12/87 = 13.8\%$
	**Europeans**	$\sim 2/87 = 2.3\%$	$\sim 6/87 = 6.9\%$

Supplemental Table S1: The setting of the “smaller” and “larger” proportions of causal variants

[a] Because the European population showed less genetic diversity than the African population, only ~2 causal loci were observed in the European samples given the smaller proportion of causal variants.

Nominal significance level = 1%																				
Europeans : Africans	0:100 (All were Africans)				20:80				50:50				80:20				100:0 (All were Europeans)			
No. of population controls	500	1000	1500	2000	500	1000	1500	2000	500	1000	1500	2000	500	1000	1500	2000	500	1000	1500	2000
TLC(1)	0.83	0.92	0.92	0.90	1.09	0.84	1.00	0.77	1.03	1.01	0.92	0.91	0.89	1.00	0.90	0.94	0.88	1.03	0.85	0.90
TLC(MAF)	0.89	0.96	0.92	0.92	0.98	0.88	0.98	0.79	1.09	1.01	1.02	0.83	1.07	1.06	0.93	0.94	1.04	0.83	0.96	1.06
TLC(PC)	0.88	1.05	0.91	0.85	0.98	0.94	1.08	1.07	0.91	1.01	1.03	0.76	0.98	0.66	0.90	0.80	1.09	0.97	0.90	0.75
TK(1)	0.68	0.86	0.86	0.78	0.98	0.88	0.92	0.96	0.85	0.82	1.05	0.88	0.79	0.70	0.75	0.89	1.04	0.83	1.07	0.71
TK(MAF)	0.72	0.83	0.62	0.69	0.80	0.83	0.74	0.81	0.57	0.64	0.83	0.70	0.80	0.45	0.60	0.62	0.64	0.68	0.75	0.49
TK(PC)	0.64	0.86	0.83	0.94	0.89	0.98	0.86	0.95	0.68	0.77	1.03	0.92	0.73	0.73	0.86	0.87	0.80	0.75	0.81	0.69
Kernel	0.91	1.01	0.93	0.81	1.08	0.75	0.84	0.99	0.76	0.88	0.79	0.78	0.67	0.87	0.50	0.78	0.81	0.80	0.79	0.78
Burden	0.93	0.90	1.04	0.89	0.83	0.94	0.95	0.80	0.98	0.70	0.91	0.73	0.63	0.52	0.61	0.53	0.86	0.85	0.85	1.02
SKAT-O	1.04	1.03	1.02	1.03	0.78	0.91	0.87	0.87	0.72	0.88	0.85	0.73	0.61	0.79	0.68	0.69	0.99	0.96	1.08	0.89
SKAT	0.87	1.02	0.95	1.01	0.88	0.83	0.81	0.98	0.64	0.89	0.75	0.74	0.81	0.90	0.58	0.81	0.96	0.92	1.02	0.98
ADA	1.03	1.01	1.04	0.89	0.79	0.87	0.88	0.96	0.72	1.03	1.05	0.88	0.63	0.87	0.65	0.80	0.96	1.05	0.88	1.06
conADA(PC)[a]	0.87	1.01	1.00	0.97	1.02	1.01	1.02	0.99	0.98	1.02	1.03	1.01	1.01	0.98	0.97	1.04	1.04	1.03	0.92	1.03
conADA(PC)[b]	0.85	1.02	0.96	1.01	1.03	1.02	1.04	1.02	1.03	1.04	1.02	0.99	0.98	1.02	1.02	1.05	1.02	1.03	0.90	1.02
Nominal significance level = 5%																				
TLC(1)	4.95	5.06	4.89	5.14	5.21	4.64	4.83	5.05	4.91	4.93	5.02	4.96	4.75	5.00	4.87	4.65	4.73	5.28	4.61	4.61
TLC(MAF)	5.18	5.00	4.98	4.74	5.12	4.47	4.91	5.23	4.71	5.07	4.98	5.15	4.93	4.88	4.95	4.91	4.68	5.25	5.07	5.12
TLC(PC)	5.02	4.89	5.09	4.97	5.26	4.77	4.67	4.93	5.00	5.08	4.81	4.86	5.20	4.49	4.54	5.06	5.21	4.86	5.26	4.81
TK(1)	4.45	4.49	4.57	4.53	4.69	4.78	4.80	4.49	4.80	4.66	4.67	4.85	4.55	4.36	4.40	4.71	4.92	4.45	4.92	4.53
TK(MAF)	4.07	4.16	4.30	4.53	4.52	4.43	4.52	4.21	4.26	4.27	4.37	4.46	4.05	3.62	4.09	4.12	4.44	4.12	4.31	3.92
TK(PC)	4.55	4.25	4.69	4.73	4.94	4.61	4.47	5.12	4.69	4.75	5.12	4.75	4.78	4.46	4.49	4.49	5.30	4.57	4.77	4.14

Kernel	4.55	4.83	4.73	5.01	4.72	4.48	4.40	4.37	4.53	4.50	4.44	4.20	3.93	4.39	3.68	4.12	4.84	4.50	4.54	4.55
Burden	4.99	5.00	5.05	4.92	5.05	4.63	4.71	4.97	4.05	4.48	4.48	4.10	3.36	3.37	3.71	3.35	4.49	5.16	5.10	5.10
SKAT-O	4.77	5.20	5.14	5.14	4.32	4.14	4.56	4.60	3.73	4.12	3.98	3.93	3.17	3.67	3.13	3.38	4.95	5.15	5.06	5.16
SKAT	4.78	5.29	4.85	5.06	4.58	4.28	4.38	4.32	3.64	4.18	3.94	4.09	3.45	4.14	3.27	3.94	5.24	4.69	4.72	4.81
ADA	4.84	5.08	4.66	5.10	4.04	4.96	4.65	4.67	3.98	4.93	5.05	4.68	3.52	4.10	3.86	4.29	5.10	5.20	4.94	5.18
conADA(PC)[a]	5.00	4.93	4.96	4.79	5.06	5.03	5.02	5.01	4.98	5.06	5.02	5.10	5.03	4.96	5.03	5.01	5.03	5.01	5.01	4.98
conADA(PC)[b]	4.92	4.86	4.93	4.75	5.05	5.07	4.97	5.04	5.03	5.07	4.98	5.09	5.04	5.02	4.98	5.02	5.02	4.99	4.98	4.95

Supplemental Table S2: Type-I error rates (%) (given the same ethnicity composition in trios and in unrelated controls)

[a] This row lists the type-I error rates of conADA(PC) with 11 candidate *P*-value truncation thresholds, $\theta_1 = 0.10,\ \theta_2 = 0.11,\ \cdots,\ \theta_{11} = 0.20$.

[b] This row lists the type-I error rates of conADA(PC) with a wider range of *P*-value truncation thresholds, i.e., $\theta_1 = 0.05,\ \theta_2 = 0.06,\ \cdots,\ \theta_{21} = 0.25$ (21 candidate *P*-value truncation thresholds).

Nominal significance level = 1%												
Europeans : Africans	20:80				50:50				80:20			
No. of population controls	500	1000	1500	2000	500	1000	1500	2000	500	1000	1500	2000
TLC(1)	0.91	1.01	0.91	0.94	0.99	0.84	0.84	0.98	0.94	1.02	1.06	1.00
TLC(MAF)	0.92	1.03	0.85	0.86	0.90	0.90	0.87	1.02	0.90	1.03	0.99	0.94
TLC(PC)	0.99	0.99	0.83	1.01	0.82	0.99	0.88	0.97	1.10	1.02	1.06	0.86
TK(1)	0.93	0.97	0.97	0.79	0.89	0.97	0.82	0.90	0.70	0.89	0.79	0.74
TK(MAF)	0.73	0.75	0.70	0.69	0.88	0.81	0.69	0.68	0.67	0.72	0.59	0.62
TK(PC)	0.93	0.97	0.98	0.75	0.89	0.97	0.87	0.98	0.84	0.98	0.88	0.85
conADA(PC)[a]	0.91	0.94	0.82	1.01	0.90	1.00	0.93	0.95	0.96	0.96	1.02	0.92
Nominal significance level = 5%												
TLC(1)	5.26	4.99	4.57	4.72	4.86	4.93	4.94	5.25	4.49	5.06	5.33	4.76
TLC(MAF)	5.12	4.92	4.82	4.88	5.04	5.05	4.81	5.18	4.78	4.87	4.97	4.83
TLC(PC)	5.22	4.97	5.31	4.86	5.04	5.04	4.72	4.93	4.86	5.21	5.12	4.96
TK(1)	4.99	4.84	4.75	4.39	4.71	4.92	4.78	4.73	4.48	4.45	4.29	4.36
TK(MAF)	4.14	4.18	4.23	4.20	4.35	4.46	4.57	4.22	4.17	4.18	4.27	3.98
TK(PC)	4.75	4.95	4.90	4.52	4.88	5.05	4.71	4.87	4.64	4.80	4.65	4.42
conADA(PC)[a]	4.95	5.01	4.99	5.02	4.70	4.55	4.76	4.58	4.53	4.68	4.70	4.45

Supplemental Table S3: Type-I error rates (%) (given a substantial difference in the source populations of trios and controls)

Given a substantial difference in the source populations of trios and controls, the five tests including Kernel, Burden, SKAT, SKAT-O, and ADA had very large type-I error rates (70%~100% rejection rates and so were excluded from the comparisons). The remaining seven valid tests were all conditioning approaches.

[a] This row lists the type-I error rates of conADA(PC) with 11 candidate *P*-value truncation thresholds, $\theta_1 = 0.10,\ \theta_2 = 0.11,\ \cdots,\ \theta_{11} = 0.20$.

Nominal significance level	1%			5%		
Europeans : Africans	20:80	50:50	80:20	20:80	50:50	80:20
TLC(1)	0.96	0.98	0.99	4.94	4.97	4.92
TLC(MAF)	1.01	1.00	0.99	4.99	5.02	4.90
TK(1)	0.88	0.89	0.85	4.72	4.70	4.69
TK(MAF)	0.74	0.77	0.78	4.38	4.31	4.32
Kernel	0.93	0.89	0.89	4.75	4.59	4.55
Burden	1.02	0.85	0.86	5.01	4.35	4.32
conADA(MAF)[a]	1.01	0.99	1.01	5.01	5.02	4.99

Supplemental Table S4: Type-I error rates (%) when only trios could be obtained (without population controls)

[a] This row lists the type-I error rates of conADA(MAF) with 11 candidate *P*-value truncation thresholds, $\theta_1 = 0.10,\ \theta_2 = 0.11,\ \cdots,\ \theta_{11} = 0.20$.

Europeans : Africans	0:100 (All were Africans)				20:80				50:50				80:20				100:0 (All were Europeans)			
No. of population controls	500	1000	1500	2000	500	1000	1500	2000	500	1000	1500	2000	500	1000	1500	2000	500	1000	1500	2000
TLC(1)	32.4	32.3	33.1	32.3	27.9	27.8	28.2	27.5	21.6	21.2	22.7	21.7	14.8	14.9	14.2	14.3	11.5	11.6	12.8	12.0
TLC(MAF)	58.6	58.9	58.1	58.7	47.5	47.9	47.7	47.6	33.6	33.7	35.1	34.3	21.9	21.9	22.0	21.9	19.5	19.7	21.2	20.1
TLC(PC)	35.4	42.9	47.2	49.1	28.5	35.5	39.3	40.2	19.1	24.2	27.5	28.9	12.5	16.2	18.1	19.2	12.5	16.1	17.4	19.3
TK(1)	16.4	16.3	16.4	16.8	13.0	12.9	12.7	13.2	8.4	7.9	8.2	7.7	4.6	4.3	4.5	4.1	4.4	4.1	4.6	4.0
TK(MAF)	44.2	43.7	44.6	44.5	31.4	31.6	32.4	31.6	17.4	17.0	16.7	16.6	9.0	8.4	8.9	9.3	13.4	13.4	13.3	14.2
TK(PC)	27.2	31.2	34.7	36.0	21.9	25.2	28.2	28.9	13.5	16.0	17.7	18.8	7.2	9.2	10.4	11.3	6.9	9.9	11.0	11.9
Kernel	61.0	70.3	76.2	79.0	46.0	55.3	61.9	64.5	26.4	35.0	39.5	42.2	15.8	21.6	25.9	28.5	22.3	31.5	36.8	41.2
Burden	67.8	73.6	76.4	79.1	56.3	62.3	66.4	69.2	38.2	44.3	48.6	51.4	22.6	27.4	30.9	33.4	25.4	30.9	35.4	37.6
SKAT-O	63.4	78.1	83.6	86.2	49.2	65.1	72.5	76.0	30.2	45.5	52.3	55.8	17.1	27.1	34.0	38.0	23.1	36.7	44.0	48.8
SKAT	48.1	67.4	75.6	79.9	32.9	51.6	61.1	64.5	17.1	31.1	38.0	42.3	9.8	18.7	25.0	28.6	15.8	31.1	37.7	43.6
ADA	72.0	83.8	85.1	87.6	62.3	78.0	80.4	81.5	47.7	67.5	69.9	72.4	26.1	44.3	50.1	51.6	27.2	38.8	42.8	44.9
conADA(PC)[a]	49.5	53.5	55.4	57.1	38.9	41.9	44.5	45.2	27.0	29.4	30.5	32.1	15.8	17.8	19.3	20.1	17.1	20.1	21.3	21.9
conADA(PC)[b]	50.0	53.1	54.5	56.6	38.4	43.0	45.4	44.7	27.4	28.8	30.6	31.8	15.7	17.9	19.6	20.3	18.4	20.1	22.3	21.8
**Nominal significance level = 5%**																				
TLC(1)	49.8	50.0	50.3	49.7	45.2	45.8	45.7	45.1	37.9	38.4	38.9	38.6	29.8	30.4	30.3	30.1	25.9	24.7	26.3	25.9
TLC(MAF)	76.0	75.3	75.3	75.9	67.0	67.0	67.5	66.8	53.7	54.1	54.1	54.2	40.8	40.9	41.2	41.4	37.9	37.9	38.6	38.0
TLC(PC)	51.1	59.1	63.3	65.5	43.9	52.1	55.6	56.7	33.6	41.6	45.3	45.7	26.0	30.6	34.5	34.8	24.7	30.7	32.9	35.9
TK(1)	32.4	32.1	32.5	32.9	27.8	26.8	27.5	27.6	20.1	20.3	20.0	20.0	13.5	12.9	14.0	13.5	13.7	13.6	14.0	13.7
TK(MAF)	65.6	65.1	66.7	65.2	51.9	52.9	53.3	52.2	34.5	34.4	34.3	34.6	23.4	23.5	23.9	23.6	30.9	31.3	31.2	31.3
TK(PC)	42.9	47.8	51.7	53.8	36.9	40.7	44.8	46.1	25.8	30.1	32.3	34.4	19.0	22.4	24.3	25.1	19.4	23.3	25.7	26.9
Kernel	79.5	86.1	89.4	91.2	66.4	75.2	79.0	81.8	46.0	56.6	60.7	63.3	34.8	41.5	46.7	50.3	44.7	53.3	58.9	62.5

Burden	82.7	85.9	88.1	88.8	73.7	78.5	81.2	82.9	58.4	65.3	67.3	69.4	42.0	47.9	51.6	53.7	44.8	50.0	53.6	55.6
SKAT-O	79.5	89.1	92.6	93.9	67.8	81.1	85.4	87.8	50.1	66.1	70.2	72.9	35.5	47.3	54.8	57.3	42.5	56.3	63.2	66.6
SKAT	67.6	83.3	89.0	91.4	51.9	71.0	78.4	81.6	33.2	51.6	59.2	62.7	24.1	37.8	45.2	49.4	34.2	51.9	59.8	64.0
ADA	85.4	92.7	93.9	94.5	78.3	89.8	91.1	92.2	67.6	84.2	85.7	86.8	45.7	66.1	71.5	73.0	46.8	60.1	64.1	64.1
conADA(PC)[a]	67.7	70.8	72.7	74.4	57.6	61.1	63.1	63.3	44.3	49.0	49.3	50.7	32.4	35.5	36.8	37.5	35.0	37.5	40.5	39.9
conADA(PC)[b]	68.1	70.9	72.8	74.8	57.4	61.9	62.3	62.7	44.9	48.7	48.4	50.9	33.0	35.7	35.7	36.3	36.1	38.9	39.3	39.4

Supplemental Table S5: Statistical power (%) given the same ethnicity composition in trios and in unrelated controls (larger proportion of causal variants; all causal variants were deleterious)

[a] This row lists the statistical power of conADA(PC) with 11 candidate *P*-value truncation thresholds, $\theta_1 = 0.10,\ \theta_2 = 0.11,\ \cdots,\ \theta_{11} = 0.20$.

[b] This row lists the statistical power of conADA(PC) with a wider range of *P*-value truncation thresholds, i.e., $\theta_1 = 0.05,\ \theta_2 = 0.06,\ \cdots,\ \theta_{21} = 0.25$ (21 candidate *P*-value truncation thresholds).

Nominal significance level = 1%																				
Europeans : Africans	0:100 (All were Africans)				20:80				50:50				80:20				100:0 (All were Europeans)			
No. of population controls	500	1000	1500	2000	500	1000	1500	2000	500	1000	1500	2000	500	1000	1500	2000	500	1000	1500	2000
TLC(1)	15.9	15.5	15.4	14.6	12.3	12.3	13.2	12.5	8.0	8.9	9.1	8.4	5.9	5.6	5.9	5.5	6.0	5.8	6.1	5.6
TLC(MAF)	21.3	21.7	21.4	20.4	16.8	16.2	16.9	17.1	10.4	11.4	11.6	11.0	7.3	7.1	7.3	7.0	8.2	7.9	7.8	7.6
TLC(PC)	51.7	61.4	67.5	69.1	41.5	50.2	55.4	58.5	27.1	35.6	40.3	41.6	18.1	23.1	26.2	27.6	20.2	26.6	28.6	30.6
TK(1)	25.4	26.0	25.9	26.0	20.0	19.2	20.0	20.0	11.0	12.1	11.9	11.6	6.5	6.6	6.6	6.2	7.6	7.7	7.8	8.4
TK(MAF)	71.8	71.0	72.2	71.6	53.1	52.9	53.3	53.9	27.7	29.4	28.8	28.0	16.8	16.4	16.9	15.7	28.9	27.8	28.7	28.4
TK(PC)	41.8	50.8	56.2	59.3	34.1	40.2	44.6	48.3	20.0	26.7	28.8	31.2	14.2	16.6	19.8	21.4	16.5	20.5	23.3	23.4
Kernel	86.5	89.8	92.2	93.8	70.9	77.7	82.2	84.9	42.0	52.5	57.1	60.2	27.9	35.8	39.6	43.6	44.5	51.9	57.0	59.5
Burden	25.6	30.4	33.0	34.5	20.0	22.8	26.0	27.8	11.5	15.3	16.4	17.6	6.5	8.3	10.4	10.6	10.0	12.1	14.1	14.6
SKAT-O	69.2	83.3	88.6	90.8	50.2	67.4	74.6	80.2	25.5	40.8	48.4	51.7	14.3	26.2	31.6	36.0	28.8	43.5	51.0	54.9
SKAT	74.3	86.6	91.2	93.4	55.2	73.6	79.1	83.8	28.8	46.3	54.3	58.4	17.0	30.7	37.0	41.7	33.5	49.4	56.1	60.0
ADA	66.5	74.3	78.2	79.6	55.7	67.0	70.8	73.1	40.4	55.2	59.5	60.9	19.8	33.0	37.9	40.8	25.4	28.4	31.6	32.7
conADA(PC)[a]	74.3	78.8	81.5	82.8	62.1	67.1	69.6	71.7	44.4	50.7	52.4	53.9	29.9	33.2	35.5	37.2	33.5	35.7	37.9	38.9
Nominal significance level = 5%																				
TLC(1)	27.6	27.8	26.8	26.0	23.9	23.1	24.4	23.9	18.0	20.0	18.7	18.1	14.3	14.6	14.9	14.4	14.6	13.9	14.4	14.3
TLC(MAF)	35.1	34.4	33.7	33.8	29.6	28.7	30.3	29.8	22.3	24.1	22.8	22.0	16.7	17.0	18.0	17.8	17.9	16.9	18.4	18.1
TLC(PC)	65.2	74.3	79.0	81.6	56.4	66.6	70.4	73.5	42.6	52.2	57.6	59.1	31.6	39.0	43.6	45.3	34.9	42.7	45.0	46.7
TK(1)	47.0	47.1	46.7	47.3	39.5	39.2	39.9	39.2	27.2	28.6	27.4	27.1	19.5	19.2	19.6	18.5	22.1	21.0	21.7	22.0
TK(MAF)	87.7	87.5	88.6	87.6	75.2	75.2	75.4	75.9	51.5	52.7	52.0	52.0	37.0	38.3	37.8	37.5	52.5	49.9	51.7	51.1
TK(PC)	61.9	70.3	75.4	77.9	53.0	60.1	65.4	68.2	37.2	45.3	49.0	50.9	30.2	34.7	39.3	40.8	34.8	39.4	43.7	44.8
Kernel	94.9	96.4	97.4	97.7	86.8	90.3	92.4	93.9	66.6	74.5	78.3	80.9	50.4	59.0	63.9	65.8	66.2	71.9	75.5	76.3

Burden	39.2	42.7	45.7	47.8	33.0	36.1	38.9	40.7	22.8	27.4	29.0	30.3	15.6	18.8	20.8	21.1	20.7	23.1	25.6	26.4
SKAT-O	85.0	92.3	95.0	96.3	69.8	83.1	87.7	90.4	45.1	62.2	69.1	71.8	31.3	45.3	52.2	55.9	50.3	62.9	69.1	71.1
SKAT	88.7	94.5	97.0	97.4	75.7	87.0	91.3	93.1	51.0	69.3	75.2	78.3	36.6	52.7	59.4	63.1	55.5	69.1	74.5	76.5
ADA	83.9	87.8	90.1	90.5	75.5	82.4	84.8	86.1	63.1	74.5	76.8	78.9	39.8	54.5	59.7	61.4	45.5	48.0	50.2	52.2
conADA(PC)[a]	87.2	89.2	91.5	91.9	78.7	82.6	84.4	85.3	65.1	70.0	71.0	72.1	49.7	53.6	56.3	56.9	53.3	55.1	58.9	58.6

Supplemental Table S6: Statistical power (%) given the same ethnicity composition in trios and in unrelated controls (larger proportion of causal variants; 50% of causal variants were deleterious and 50% were protective)

[a] This row lists the statistical power of conADA(PC) with 11 candidate *P*-value truncation thresholds, $\theta_1 = 0.10,\ \theta_2 = 0.11,\ \cdots,\ \theta_{11} = 0.20$.

Nominal significance level = 1%																				
**Europeans : Africans**	0:100 (All were Africans)				20:80				50:50				80:20				100:0 (All were Europeans)			
No. of population controls	500	1000	1500	2000	500	1000	1500	2000	500	1000	1500	2000	500	1000	1500	2000	500	1000	1500	2000
TLC(1)	4.5	4.6	4.7	4.7	3.9	3.7	3.6	3.7	2.9	3.0	2.8	2.6	2.1	2.8	2.0	1.9	2.4	2.4	2.3	2.1
TLC(MAF)	8.0	8.3	8.1	7.8	6.2	5.6	6.1	5.7	4.1	3.9	4.3	3.7	2.7	3.7	2.6	2.7	3.0	3.1	3.5	2.8
TLC(PC)	6.5	8.2	9.0	10.1	4.6	6.3	6.8	6.9	2.8	3.7	4.5	4.6	2.4	2.9	3.1	3.1	2.7	3.3	3.4	3.9
TK(1)	3.0	3.3	3.3	3.0	2.9	2.8	2.7	2.4	1.7	1.8	1.8	1.9	1.5	1.3	1.2	1.1	1.4	1.7	1.4	1.6
TK(MAF)	8.7	9.4	8.7	9.2	6.0	6.1	5.7	5.8	2.7	3.2	3.1	2.8	1.7	2.0	1.9	1.9	3.1	3.1	3.0	3.2
TK(PC)	5.6	7.5	8.5	8.8	4.8	5.5	6.0	6.5	2.8	3.5	3.9	4.0	2.1	2.4	2.7	3.0	2.5	3.3	3.5	3.8
Kernel	14.9	19.1	22.6	24.5	9.3	12.6	14.3	16.0	4.4	6.3	7.6	8.7	2.9	4.4	5.0	5.6	5.4	7.8	9.1	10.2
Burden	9.8	12.1	13.9	15.0	6.9	8.4	9.7	10.2	3.6	4.8	5.7	5.6	2.2	3.4	2.9	3.4	3.3	4.5	5.2	5.5
SKAT-O	12.2	18.8	23.6	25.6	7.7	12.5	15.4	16.9	3.6	6.3	8.0	9.2	2.8	4.2	4.9	5.6	4.3	7.5	9.4	10.9
SKAT	10.7	17.6	22.7	25.3	6.2	11.4	14.1	16.0	3.0	5.5	7.3	8.4	2.6	3.8	4.7	5.5	4.4	7.8	9.7	11.6
ADA	15.1	23.3	27.0	28.4	10.6	18.4	20.7	20.6	6.4	13.0	14.8	14.7	3.4	7.5	8.5	9.2	5.3	8.2	10.1	11.0
conADA(PC)[a]	12.0	13.9	15.1	15.8	8.7	10.3	10.1	11.1	5.3	6.4	7.4	7.2	3.6	4.8	4.7	5.0	4.9	5.9	6.2	6.0
**Nominal significance level = 5%**																				
TLC(1)	12.8	13.8	13.4	12.3	11.7	11.0	11.5	11.3	10.1	9.6	10.6	9.5	8.1	9.0	8.3	7.9	8.4	8.4	8.8	8.5
TLC(MAF)	19.8	20.2	20.3	19.7	16.5	16.1	16.8	15.8	12.8	12.4	13.3	12.2	9.7	10.5	10.2	9.6	10.2	10.8	11.1	10.8
TLC(PC)	15.3	18.3	19.6	21.3	12.3	14.7	16.0	16.8	10.0	11.5	12.8	12.6	8.5	9.5	10.3	10.8	9.4	10.9	11.0	12.0
TK(1)	11.3	10.4	10.8	10.7	9.8	9.3	9.4	9.2	7.4	7.2	8.1	7.6	6.6	6.7	6.8	6.8	6.7	7.4	7.0	7.3
TK(MAF)	23.5	22.6	22.9	22.6	16.8	16.8	16.3	17.0	10.8	10.9	11.5	10.9	7.9	9.1	8.5	8.9	11.7	12.2	11.4	11.5
TK(PC)	14.6	17.4	19.2	20.2	12.8	14.6	15.3	16.4	9.1	10.8	11.9	12.0	8.2	9.5	10.2	10.7	9.0	10.9	12.0	12.2
Kernel	32.5	37.4	42.7	43.7	22.9	27.8	30.2	32.9	14.3	17.4	20.0	21.3	10.4	14.4	14.9	16.9	16.4	20.8	22.8	25.3

Burden	22.3	25.6	28.3	29.2	17.7	19.9	22.1	22.7	12.0	13.8	15.5	15.9	7.8	10.4	10.2	10.4	11.0	12.8	14.7	14.6
SKAT-O	26.4	35.1	41.5	43.3	18.8	25.6	30.3	32.7	11.4	16.3	19.3	21.1	8.8	12.5	13.5	14.9	13.5	19.0	22.2	24.5
SKAT	24.7	35.7	42.4	43.8	16.8	25.4	29.6	32.2	10.3	15.7	18.6	20.7	8.5	12.7	14.0	16.1	13.6	20.5	23.1	26.3
ADA	31.2	42.1	46.2	48.2	24.0	35.6	39.0	40.1	18.2	30.0	31.9	32.3	11.0	20.2	22.7	23.6	14.8	21.6	23.3	25.0
conADA(PC)[a]	26.6	29.2	30.4	31.4	20.2	23.0	23.5	23.8	15.4	17.1	19.2	18.4	12.0	14.0	14.1	14.9	14.2	16.3	16.8	17.0

Supplemental Table S7: Statistical power (%) given the same ethnicity composition in trios and in unrelated controls (smaller proportion of causal variants; all causal variants were deleterious)

[a] This row lists the statistical power of conADA(PC) with 11 candidate *P*-value truncation thresholds, $\theta_1 = 0.10,\ \theta_2 = 0.11,\ \cdots,\ \theta_{11} = 0.20$.

Nominal significance level = 1%																				
Europeans : Africans	0:100 (All were Africans)				20:80				50:50				80:20				100:0 (All were Europeans)			
No. of population controls	500	1000	1500	2000	500	1000	1500	2000	500	1000	1500	2000	500	1000	1500	2000	500	1000	1500	2000
TLC(1)	3.8	4.3	3.8	4.2	3.4	3.0	3.5	3.2	2.5	2.5	2.2	2.4	1.9	1.8	1.6	1.9	2.1	2.2	2.3	2.1
TLC(MAF)	5.4	6.0	5.2	6.1	4.0	3.7	4.3	3.9	2.9	3.1	2.6	2.9	2.2	2.1	2.1	2.2	2.5	2.7	2.6	2.4
TLC(PC)	13.3	17.8	19.2	21.6	9.8	11.9	14.3	14.9	5.4	7.2	7.8	8.5	3.8	4.7	5.5	5.7	4.8	6.2	6.8	6.8
TK(1)	5.7	6.7	5.6	5.8	4.9	4.7	4.8	4.4	2.8	2.9	3.0	2.6	2.0	2.3	2.0	1.9	2.6	2.5	2.8	2.6
TK(MAF)	21.9	22.2	20.9	21.3	13.1	12.5	12.6	12.2	6.3	6.2	5.8	5.7	3.7	4.1	3.6	3.5	7.3	7.3	7.6	7.0
TK(PC)	14.3	19.1	21.7	23.5	10.1	13.0	15.2	16.0	5.5	6.9	8.0	9.0	4.0	5.3	6.7	6.5	6.1	7.3	8.2	8.7
Kernel	34.6	42.4	46.8	49.4	21.8	26.6	30.7	32.6	10.0	12.7	14.5	16.2	5.8	8.6	9.6	10.4	13.1	16.6	18.2	19.8
Burden	6.7	9.3	9.1	10.4	4.9	5.6	6.8	6.8	2.8	3.5	3.4	3.9	1.6	2.0	2.0	2.3	3.1	3.6	4.5	4.4
SKAT-O	20.5	33.7	39.3	43.4	12.2	19.2	24.8	27.2	5.0	8.8	10.9	13.0	3.0	6.0	7.0	7.7	7.6	12.8	15.8	17.7
SKAT	24.1	38.5	45.6	48.9	14.2	23.4	29.1	31.6	6.3	10.5	13.3	15.8	4.0	7.6	8.9	10.0	9.3	15.4	18.5	20.5
ADA	18.0	25.4	28.0	30.1	13.4	19.1	22.8	22.4	8.1	13.3	15.2	16.2	3.8	7.1	8.5	9.3	6.9	8.5	9.0	9.1
conADA(PC)[a]	28.0	32.4	35.1	35.6	19.5	22.0	24.3	25.2	12.1	13.4	15.0	15.5	7.1	8.8	9.7	9.8	10.1	11.4	12.1	12.2
Nominal significance level = 5%																				
TLC(1)	11.1	12.0	10.4	12.1	9.8	9.7	10.4	9.3	8.5	8.3	7.9	7.6	7.1	7.2	7.1	7.4	8.0	8.0	8.2	7.7
TLC(MAF)	14.5	15.0	13.9	14.3	11.8	11.4	12.1	11.2	9.9	9.7	9.0	9.2	7.8	8.0	7.8	8.1	9.2	8.9	9.3	8.9
TLC(PC)	25.9	32.3	34.6	36.4	21.0	24.9	27.2	28.1	13.6	17.0	18.6	19.9	11.3	13.4	14.0	14.7	13.0	15.2	16.9	17.0
TK(1)	16.9	17.2	16.5	17.4	14.3	13.8	14.5	14.0	10.5	10.9	10.6	10.1	8.6	8.6	8.3	8.1	9.9	9.8	9.7	10.0
TK(MAF)	43.1	42.6	41.6	42.3	30.6	29.5	29.8	29.7	18.6	18.1	17.5	17.2	13.2	12.8	12.8	13.1	20.3	20.4	19.5	19.6
TK(PC)	29.6	35.5	39.4	41.9	23.1	27.7	31.0	32.1	15.4	18.5	20.1	21.4	12.5	15.1	17.4	17.7	15.8	18.6	19.3	20.5
Kernel	56.3	63.1	67.0	67.8	42.1	47.4	52.0	53.3	25.1	29.0	31.8	33.6	18.2	22.4	23.1	24.7	28.0	31.8	34.4	36.2

Burden	16.1	18.7	19.1	21.0	12.9	13.7	15.7	15.4	9.1	9.8	10.3	11.1	6.5	6.6	7.3	8.3	9.8	11.0	11.3	11.5
SKAT-O	37.7	52.7	58.4	60.9	26.5	36.4	42.6	44.8	15.1	20.4	24.4	26.8	10.2	15.7	17.0	18.9	19.3	26.7	29.2	31.7
SKAT	44.2	59.3	65.2	67.5	31.3	42.8	49.2	52.2	18.0	24.8	29.8	32.1	13.0	19.5	20.9	23.6	23.1	30.6	34.0	36.8
ADA	36.0	44.0	46.8	47.1	29.9	36.3	39.6	40.3	21.3	28.6	31.3	32.5	12.8	18.4	21.1	22.7	17.7	19.7	20.4	21.9
conADA(PC)[a]	47.2	51.3	54.5	54.4	37.3	40.1	43.2	43.8	26.7	29.4	30.9	31.7	19.1	21.3	22.7	23.5	23.0	25.2	25.6	25.9

Supplemental Table S8: Statistical power (%) given the same ethnicity composition in trios and in unrelated controls (smaller proportion of causal variants; 50% of causal variants were deleterious and 50% were protective)

[a] This row lists the statistical power of conADA(PC) with 11 candidate *P*-value truncation thresholds, $\theta_1 = 0.10,\ \theta_2 = 0.11,\ \cdots,\ \theta_{11} = 0.20$.

	Type I error rates (%)				Statistical power (%)			
**Significance level**	1%		5%		1%		5%	
Linkage disequilibrium	Lower [b]	Higher [b]	Lower	Higher	Lower	Higher	Lower	Higher
TLC(PC)	0.91%	0.79%	4.58%	4.90%	3.54%	3.51%	10.14%	9.80%
TK(PC)	0.69%	0.64%	4.50%	4.53%	3.88%	4.70%	13.99%	14.03%
conADA(PC)[a]	0.96%	0.84%	4.99%	4.97%	36.41%	27.34%	62.08%	49.83%

Supplemental Table S9: Type I error rates (%) and statistical power (%) under admixture scenario (larger proportion of causal variants; 50% of causal variants were deleterious and 50% were protective)

[a] This row lists the Type I error rates or statistical power of conADA(PC) with 11 candidate *P*-value truncation thresholds, $\theta_1 = 0.10,\ \theta_2 = 0.11,\ \cdots,\ \theta_{11} = 0.20$. The *P*-value of conADA(PC) was obtained with the sequential Monte Carlo permutation (the minimum and maximum numbers of permutations were set as 100 and 10000, respectively).

[b] In the 10,000 simulation data sets, the average r^2 between any two variants ranged from 0.005 to 0.035, with a median of 0.0093. The results of the “Lower” linkage disequilibrium (LD) group were based on the 5,000 simulation data sets with the average r^2 <= 0.0093, whereas the results of the “Higher” LD group were based on the 5,000 simulation data sets with the average r^2 > 0.0093.
